# ‘I Think That Is a Big Step From Here to There’: Exploring the Views of Service Providers About Delivering Drug‐ and Alcohol‐Related Health Services to Aboriginal People in Rural New South Wales

**DOI:** 10.1111/dar.14096

**Published:** 2025-06-08

**Authors:** Raechel Wallace, Simon Clay, Anthony Shakeshaft, Sara Farnbach

**Affiliations:** ^1^ National Drug and Alcohol Research Centre UNSW Sydney Sydney Australia; ^2^ Network of Alcohol and Other Drugs Sydney Australia; ^3^ Poche Centre for Indigenous Health, University of Queensland Brisbane Australia

**Keywords:** aboriginal, Australia, health services accessibility, primary healthcare, substance‐related disorders

## Abstract

**Introduction:**

The higher rates of alcohol and other drug (AOD)‐related harms in rural compared to metropolitan areas demonstrate the need for consideration of rural AOD health service delivery. We aim to explore the experiences of health service providers around delivering AOD services to Aboriginal and Torres Strait Islander (hereafter, Aboriginal) people in rural New South Wales (NSW) and opportunities to optimise services.

**Methods:**

Open‐ended, semi‐structured interviews or written responses were conducted with 18 Aboriginal and non‐Aboriginal service providers at Aboriginal community‐controlled health services, mainstream services or experts who work with Aboriginal people around AOD in rural NSW. Data were thematically analysed, led by an Aboriginal researcher.

**Results:**

Five themes were identified: (i) the role of family and community; (ii) the role of health services (particularly Aboriginal Community Controlled Health Services) in building cultural connections with clients; (iii) inconsistent referral and communication procedures impact the continuity of care; (iv) difficulties hiring, training and retaining Aboriginal workers; and (v) the role of technology in enhancing service access.

**Discussion and Conclusions:**

Key opportunities to improve the delivery of health service to Aboriginal people in rural NSW include programmes to drive culturally meaningful care, in both Aboriginal and non‐Aboriginal services, systemic changes to increase access to AOD training among Aboriginal workers, and better remuneration. Shared clinical records and telehealth may enhance service delivery by increasing service access and referral processes; however, Aboriginal community leadership and cultural safety are crucial for safe design and delivery.

**Summary**

- There are opportunities for Aboriginal and non‐Aboriginal services to deliver culturally meaningful care.
- Shared health records and telehealth services may increase service access but should be designed by Aboriginal people to build trust, safety and ensure therapeutic relationships are maintained.
- Better access to training and development, remuneration and funding for longer contracts of dedicated drug and alcohol workers within Aboriginal Community Controlled Health Services may help to continue to build the Aboriginal workforce.

## 1 Introduction

Although rural and regional communities experience more alcohol and other drugs (AOD) related harms such as risky alcohol consumption [1] and drug‐related hospitalisations [2] compared to metropolitan communities, access to AOD‐related health services is often more limited [3, 4]. The smooth operation and coordination between a range of primary and tertiary health services to complete assessment, counselling, case management, withdrawal management or rehabilitation is crucial to addressing these harms. However, there are limited available tertiary services in rural areas, placing greater importance on primary and community‐based services to deliver this care. Among Aboriginal and Torres Strait Islander (hereafter, Aboriginal) people, access to health services can be further hampered by cultural barriers [5] and limited cultural safety [6, 7]. With approximately 60% of Aboriginal people living outside major cities [8] where most services and clinicians are located, the specific need for considered service delivery approaches to Aboriginal people in rural and regional areas is needed.

Aboriginal people have demonstrated longstanding resilience and strength for over 60,000 years [9], with traditional approaches to maintaining health including complex kinship and cultural systems [9]. Since the 1970s, a national network of Aboriginal‐controlled, culturally specific health services has been delivered by Aboriginal Community Controlled Health Organisations (ACCHO). The ongoing impacts of colonisation continue to impact health outcomes among Aboriginal people who, despite being 1.4 times less likely to have consumed or had any alcohol in the last 12 months compared to non‐Indigenous Australians [8], experience greater harms from AOD [10] and have more cultural and logistical barriers to accessing support services [5, 6, 7, 11]. Approaches suggested in the literature to address these barriers include more locally based inpatient services [12], flexible service offerings [12] and efforts to improve cultural security in all health services [11]. Recent work has also identified promising ways to integrate cultural knowledge and healing practices with Western medical approaches via enhanced interpersonal processes and service‐wide strategies such as employing Aboriginal workers and delivering services at home [13]. This unique set of conditions surrounding Aboriginal people in rural areas demonstrates the need for health services for AOD‐related care that are tailored to these specific circumstances.

### 1.1 Aim

To explore the experiences of health service providers about delivering AOD‐related services to Aboriginal people in rural New South Wales (NSW) and how to optimise service delivery.

## 2 Methods

### 2.1 Approach

This qualitative study used semi‐structured, open‐ended interviews to explore service providers' views. Data were analysed using thematic analysis, with transcripts coded into key concepts and ideas to identify themes relevant to the research aim [14].

### 2.2 Participants

Eligible participants were health service providers delivering AOD related care to Aboriginal people in rural NSW, as they have current experience relevant to the research aim. As such, those delivering AOD services at ACCHOs (e.g., Aboriginal medical service, Aboriginal primary healthcare organisations or Aboriginal residential rehabilitation organisations), or who regularly deliver AOD care to Aboriginal people in non‐ACCHO primary healthcare services in rural or regional NSW, were eligible. Managers at ACCHOs were also eligible. Participants also included Board Members of Aboriginal Corporation Drug and Alcohol Network (ACDAN), a peak body supporting NSW Aboriginal AOD workers. These Board Members were included because they have experience delivering AOD related care to Aboriginal people and were familiar with AOD service delivery in rural NSW.

### 2.3 Aboriginal Leadership, Governance and Reflexivity

This study arose from discussions with representatives from Aboriginal experts who work in the AOD sector and voiced their concerns about gaps in the AOD system and accompanying challenges in delivering care to Aboriginal people. RW led data collection, analysis and reporting. RW is an Aboriginal AOD expert with service management, workforce and research skills who has worked in the sector for 19 years. Analysis was supported by SC and SF, both of whom are non‐Aboriginal and experienced qualitative researchers. SF has worked with Aboriginal communities for 10 years. AS contributed to the formative work and commented on the manuscript. He is a non‐Aboriginal researcher in the AOD field committed to culturally competent research practices and fostering two‐way learning approaches.

### 2.4 Sampling Strategy

RW identified rural or regional ACCHO and non‐ACCHO AOD or primary healthcare services whom, based on her 19 years' experience working in the NSW AOD sector, regularly deliver AOD‐related care to Aboriginal clients. A snowballing method was also used where initial participants suggested other potentially relevant and eligible services to RW, who contacted them to inform them about the study [15]. RW approached Chief Executive Officers or research managers at these services who were authorised to approve the study to inform them about the study and participant eligibility. Potential participants at non‐ACCHOs self‐reported if they regularly deliver AOD care to Aboriginal people in rural NSW. Due to resource and logistical limitations, we were unable to invite all potentially relevant services in rural NSW. In total, eight services were approached and six took part from five areas in NSW classified as large, medium or small rural towns or very remote communities [16]. Managers provided consent for the study at their service and circulated the Participant Information Sheet and Consent Form to potentially eligible staff. If interested, individuals contacted RW to arrange a time to complete informed consent, and then RW organised an interview. The study concept was presented at an ACDAN quarterly board meeting, and members endorsed involvement. All ACDAN Board Members were invited to take part.

### 2.5 Data Collection Techniques

Interviews were conducted by RW or RW and SF via phone, Microsoft Teams or in‐person between July and December 2022. Interviews were audio recorded then transcribed verbatim by a professional transcription service. An interview guide was developed based on the literature and RW's experience in the field and comprised questions about current pathways through AOD services, gaps in pathways and solutions to address these gaps. In response to a request from ACDAN board members, the interview guide was emailed to members who wanted to participate but were unable to do an interview so they could provide written answers to RW.

### 2.6 Data Analysis

Interviews and written responses were inductively and deductively coded in NVivo using thematic analysis [14]. RW read all transcripts and assigned codes to the key concepts and common experiences in the data. This was an iterative process, meaning that the analysis of previous transcripts informed the next ones [14]. Written responses were analysed alongside the interview data to identify any overlapping or unique themes. The findings from each data set enriched the analysis of the other. These codes and data were then jointly reviewed by SC, SF and RW and themes identified. Pseudonyms were used in place of participants' names.

### 2.7 Ethics

This research was presented to and endorsed by ACDAN and approved by the Aboriginal Health and Medical Research Council (1887/21) as well as the UNSW Ethics Committee (1887 21). It is conducted using the principles outlined in consolidated criteria for strengthening reporting of health research involving Indigenous peoples [17] and common standards for qualitative research [18].

## 3 Results

Eighteen participants from six different ACCHOs and non‐ACCHO primary healthcare services and ACDAN Board Members from rural NSW completed interviews or written responses. Of these, eight were managers or in shared clinical and managerial roles, five worked as clinicians and five were ACDAN members. Nine were Aboriginal, 10 were men and eight were women. Participants' qualifications ranged from vocational training to university qualifications. All had at least 2 years working in rural NSW health services. Seven completed small group interviews (via Microsoft Teams), six completed one‐on‐one interviews (one face‐to‐face and five by phone) and five provided written responses.

Five themes were developed from the analysis of participants' interviews: (i) the role of family and community; (ii) the important role of health services (particularly Aboriginal Community Controlled Health Services) in building cultural connections with clients; (iii) inconsistent referral and communication procedures impact continuity of care; (iv) difficulties hiring, training and retaining Aboriginal workers; and (v) the role of technology in enhancing service access.

### 3.1 The Role of Family and Community

Participants emphasised the importance of family and community and how these connections enhanced engagement. Clients typically had a catalyst moment that led them to contact health services for AOD‐related care. These moments tended to centre on the desire to recover damaged relationships or revitalise lost family or community connections. Participants reported that family members have an important positive influence on Aboriginal clients and want to support clients; however, problems sometimes arose when family members did not have access to information or resources on how to best support a loved one around their AOD use. For instance, this worker of a service that delivers an abstinence‐focused model reported:

‘We've had parents come … [and] pick them up and take them to the club for a feed and have a beer [and ask], ‘Why can't you have a beer, son?’ You know, fathers have a drink with their sons. It's getting through some of that thinking. It's just a misunderstanding. It's not that they do anything wrong; they just need more education.’ Jack

Participants reported their efforts to address this issue by working with family members to inform them about the changes they could expect from their family member after they left AOD rehabilitation services.

Community was highlighted as a vital aspect of the AOD treatment and posttreatment process as it was a source of vital support to Aboriginal people, providing social and cultural connections and logistical support. For instance, it was common for individuals to have no secure housing to return to posttreatment, so community or family members would often open up their homes to ensure the person had somewhere to stay:

‘In the Aboriginal community, unless you're really, really crazy on the drugs and got mental health problems, you don't really end up homeless. There's always someone that will take you in, one of the mob will. So again, it's a hard step, but housing is one of our biggest problems.’ Jack

### 3.2 The Important Role of Health Services (Particularly Aboriginal Community Controlled Health Services) in Building Cultural Connections With Clients

Culture was viewed as central to optimise service delivery within services, with most participants highlighting the strengths of incorporating culture‐based healing and spiritual practices into care:

‘Culture is a very powerful tool we pull out of the toolbox, and culture, identity, and normative kind‐of proud lifestyles are things that really motivate Aboriginal people, and they can ground them.’ Matthew

Many ACCHOs in NSW have a cultural aspect of service delivery, such as programs that create links with Elders and cultural dance. These participants described how these services were more than a health service: they were places where individuals could reconnect with their culture and Aboriginal identity. This holistic, culturally‐informed approach to supporting clients and their families was crucial to improving well‐being. Participants viewed these cultural programs as a necessary part of service delivery and was particularly beneficial for clients who did not have a strong connection to their cultural and ancestorial heritage prior to attending this service:

‘When [clients] were doing a lot of cultural stuff [at the clinic], when they come back, they're different people, you know? But I often think, “Do people have to go to rehab to be able to connect with culture? They should be able to do that in their own backyard”.’ Sharon

Participants highlighted how many of the Aboriginal workers at their AOD services would draw upon their cultural skills and connections during their work. This would generate a more trusting connection with the community, leading to increased access and ongoing engagement with the service. Participants emphasised how better recognition of Aboriginal workers as expert knowledge holders is needed in health policies and by employers.

Participants also discussed the importance of clients building healthy connections with people with similar cultural backgrounds and lived experiences with AOD. These community connections were viewed as essential to individuals' ability to achieve their health goals. These connections sometimes occurred through peer support programmes, such as 12‐step Alcoholics Anonymous or SMART programmes [19].

### 3.3 Inconsistent Referral and Communication Procedures Impact on Continuity of Care

Participants reported that the policies and procedures that guide health services sometimes lacked clarity and did not adequately cover the full process of clients entering, using and leaving both outpatient and residential services for AOD‐related care. This often resulted in poor coordination between services, which negatively impacted ongoing client engagement. For example, many participants stated that referrals were often incomplete and insufficient time was allocated to planning transitions from one service to another. The negative consequence of incomplete referrals was particularly acute in rural areas, where it is harder to access specialist services compared to the cities and exacerbated by the multiple priorities often faced by people accessing AOD‐related care, including mental illness, complex families, difficulties securing employment and housing, which were not always met by the system:

‘[Coordinating with other services] helps the individual. [It is also good] knowing they have something to go back to as well. Otherwise, yeah, how do you support somebody to return to a small community? I find one of the problems or one of the difficulties is finding out what services actually service those [rural] communities so we can communicate with them, set up a referrals process, and hope to get that person continuous support.’ Joanne

To overcome this barrier, participants suggested integrating client data systems so that client data could be recorded and accessed by treating clinicians at different services. This system could record basic clinical information including health assessments, client goals, outcome monitoring, treatment plans and referrals. Such a system may improve the flow of clients through the system and have additional benefits to clients by reducing the burden on them to repeat their story each time they use a service:

‘[We need to make services] as integrated and as seamless and flawless as possible, because the last thing [clients] want to do is tell their story 15 times. [Clinicians] can see one person, your [inter‐service] systems talk, they put your notes and your GP [into the system], now [any clinician] can see what they've actioned from elsewhere, and it all has to be like a well‐oiled machine and not in silos.’ Joseph

Participants outlined how certain logistical and administrative barriers would need to be overcome if a shared data system across services was to be established. They also emphasised the need for clinicians and clients to be involved in the development of this process to ensure safety and confidentiality were maintained.

To overcome inadequate policies and procedures, many participants described the informal coordination techniques they had developed, such as completing ‘warm referrals’ where they took clients to the next service to directly introduce them to the clinicians there. These informal service coordination methods yielded a range of benefits, including strengthening social and professional connections between services, creating pathways between and across health services and improving access to holistic care among clients with complex needs. Participants also developed processes to stay in contact with clients' posttreatment via home visits, phone calls or Zoom to keep them engaged and maintain a pathway to an AOD service. Participants reported that having a known staff member provide ongoing support to a client after discharge was highly beneficial because it provided a clear avenue for the client to reengage with the AOD system if they needed it:

‘It doesn't matter if they start using again. We talk to them through that; we'll still keep in contact. It's not like once they start using or drinking we wipe our hands of them. We'll keep working with them and maybe bring them back through rehab or whatever, refer them somewhere to another service … But we will still keep in contact with them.’ Jack

### 3.4 Difficulties Hiring, Training and Retaining Aboriginal Workers

Hiring, training and retaining Aboriginal workers was difficult for many participants and their health services. This issue was compounded by the limited number of appropriately skilled AOD workers (particularly Aboriginal Health Practitioners) in rural areas. These participants discussed how low remuneration, high levels of burn‐out and limited training opportunities were the leading causes for the rapid turnover of staff and the inability to fill vacancies in a timely fashion.

Many participants reported that training and skill development opportunities around AOD skills were limited and typically difficult to access, especially in rural areas, resulting in many workers taking other professional opportunities to build their careers and skills outside AOD. Participants in managerial positions reported difficulties sending clinicians to AOD or other training because of challenges backfilling their role to ensure essential services could be met.

‘We were having a look just a couple of weeks ago, me and the HR officer, and one of the TAFEs were offering the Cert IV Drug and Alcohol and it was two days a week out of the office for the next 12 months … Our catchment area's quite huge, and they're outreaching two, three days a week, and then they've got their local clients here. There's a men's group, we've got to start up the women's group, and then having the ability to then be in the office and put their notes on and do all their admin stuff, it wasn't feasible [for them to do the Cert IV].’ Belinda

Manager participants recounted frustrating instances of hiring Aboriginal workers into AOD roles with minimal experience working in AOD and providing them with AOD training, only to have these workers receive job offers with higher incomes, more training and development opportunities and better job security (e.g., government roles), which they invariably took. Participants understood the motivations of workers to take these other jobs, however, they also stressed that this staffing issue was the product of a poorly structured health system that did not provide suitable resources and funding to develop and train AOD workers:

‘Even when I did my Drug and Alcohol Nursing [certificate], it was $12,000 for a 12‐month course, and nobody's got that money. I'm still paying it off … When you say to someone [how much it costs to do training], it's kind‐of like, “No, I can't do that, I can't afford that”.’ Belinda

Community AOD services typically operate on short‐term funding and are required to regularly undergo the administrative‐intense process of requesting and managing existing funding, which requires them to develop detailed responses to tenders and report against multiple outcome indicators to different funding providers. Participants spoke about how smaller services, particularly ACCHOs, were adversely affected by this process, particularly in relation to service delivery and workforce operations. For instance, short‐term funding was often renewed at the last minute, which led to workforce shortages across AOD service types and regions because clinics had problems recruiting and retaining appropriately skilled workers. Consequently, many good workers sought out more stable employment:

‘That's one of the things—you get 12 months of funding. You don't hear that the funding is coming through until a couple of weeks before it's due to end, and by that time, half your good staff have racked off … [and] gone and got other jobs. There's no security for people.’ Leanne

### 3.5 The Role of Technology in Enhancing Service Access

Participants provided examples of how the use of digital technology, like telehealth and online access to group sessions, facilitated health service access and improved health outcomes. The recent increased availability of telehealth was viewed positively by some who reported that it created service access where it would otherwise not be available. However, others reported that it was difficult for people to build rapport and develop relationships via telehealth, which could negatively impact therapeutic relationships. Participants were wary of clinicians and clients becoming over reliant on telehealth as this could lead to missed important opportunities to effectively engage with Aboriginal people in rural areas.

Technology was reported to enhance access to services among people who were waiting for a bed in residential rehabilitation service, by a service that offered virtual access to daily cultural group activities for people on their wait list. This was viewed as a positive use of technology because it enhanced engagement when access opportunities were limited and helped to build rapport between future clients and clinicians.

## 4 Discussion

The study, which was led by an Aboriginal service delivery expert and researcher, highlights the complexity of the health system in rural NSW regarding AOD and identifies opportunities to enhance the delivery of AOD‐related care to Aboriginal people in rural NSW.

### 4.1 The Influence of Sociocultural Factors

Participants in this study echo previous research highlighting the importance of incorporating cultural and spiritual practices into non‐ACCHO and ACCHO health service delivery to create positive health outcomes [11]. For instance, a recent systematic review [20] found that most of the published research focused on the effect of culture was positively correlated with improved physical health, social and emotional wellbeing outcomes and reduced risk‐taking behaviours. These benefits were thought to arise from connecting with Country, language, knowledge, beliefs, kinship and family [20], with our participants also adding the importance of connecting with Elders and having opportunities for cultural expression, to improve the AOD service delivery.

Further, AOD use and the delivery of services should not be considered in isolation from the historical, socioeconomic and political context for Aboriginal people in which they occur [11]. An example of an initiative that centralises culture in service delivery was led and delivered by the Aboriginal Drug and Alcohol Residential Rehabilitation Network (ADARRN—a network of Aboriginal residential rehabilitation services located throughout Australia focused on the needs of Aboriginal people) [21]. Over recent years, ADARRN has demonstrated an approach to delivering standardised, culturally meaningful care across multiple services that can be tailored to individual services, thereby allowing for local flexibility.

Approaches to enhance cultural safety in service delivery, such as the ADARRN model, should be considered elsewhere, including in mainstream (non‐ACCHO) services, to ensure programs, services and policies are delivered in a way that respects and acknowledges cultural difference [11]. Prior work has shown that specific actions likely enhance the acceptability and accessibility of mainstream services include culturally sensitive communication and a providing a welcoming physical environment [22]. However, steps that mainstream services can take to integrate cultural safety remain unclear in the literature. One example from mainstream NSW AOD services involved developing a treatment guideline for working with Aboriginal people in non‐Aboriginal services [23]. In this work, individual services were supported with tailored implementation support, and pilot work demonstrated this approach was both feasible and acceptable [24], and appeared to improve the competence of service delivery [25]. However, caution should be taken when taking this approach as it should be led by local cultural experts to ensure practice changes are suitable and culturally competent and not a tick‐box exercise.

Participants described that the cultural skills of Aboriginal workers, particularly those at ACCHOs were crucial because they built connections and generated trust with individuals and the community, which is important in delivering AOD related care. Prior research has described how strong interpersonal relationships can generate trust, via a process of yarning, humour and connection, which builds a common ground, ultimately facilitating trust [13]. Building such connections requires skilled individuals who are able to work ‘bi‐culturally’ by blending cultural knowledge with Western medical knowledge [13]. As well as often employing individuals with these cultural skills, research has also demonstrated that the operational aspects of ACCHOs such as governance, employment and training enhance the broader health system [26]. These improvements are driven via policy that reduces alcohol‐related presentations at health services, employment opportunities for local Aboriginal workers and cultural safety training for non‐Aboriginal health workers [26]. Together, these results indicate that to improve AOD related services in rural NSW to Aboriginal people, both ACCHO and non‐Aboriginal services should be encouraged to continue to focus on the delivery of culturally safe services and the cultural and therapeutic skills of ACCHOs and Aboriginal worker should be acknowledged and fostered.

### 4.2 Recruiting, Training and Retaining Aboriginal Alcohol and Other Drug Workers

The crucial role of Aboriginal AOD workers in the delivery of AOD health services and the difficulties in recruiting, training and retaining appropriately skilled workers was identified by participants. According to our participants, these difficulties stem from low pay grades, few training and development opportunities, and a lack of job security in ACCHOs and AOD roles, compared to other health‐related positions. The crucial skills held by Aboriginal workers in connecting with individuals and as important cultural knowledge holders were also identified. While some workers may find drawing on their cultural skills motivating, as it can enhance job satisfaction and help inform their practice [27], difficulties navigating the dual roles as health worker and community member have been identified, as it can lead to work‐life imbalance and contribute to burn‐out [28, 29]. Also potentially contributing to burn‐out, research profiling the NSW AOD workforce demonstrated that almost all of the 51 participating Aboriginal AOD workers reported having work expectations that are too high and limited supervision arrangements [30]. Given shortages in Aboriginal health workers [31] and that many Aboriginal workers are reportedly drawn to other positions due to the superior work conditions and opportunities for professional development, the Aboriginal AOD workforce could be grown via systemic changes to enhance training opportunities [27, 31] and improve remuneration [27]. For instance, greater access to existing AOD training programs delivered by the Aboriginal Health and Medical Research Council of NSW [32] or programs carefully designed and delivered in the university setting provides opportunities to continue building the Aboriginal AOD workforce [33]. For these programs to succeed, they need to be sustainably funded.

### 4.3 Healthcare Service Delivery

Participants highlighted how digital technology could be optimised or utilised to improve service delivery and increase access to health services among clients in rural areas. To improve communication and referral between services, participants suggested integrating client data systems, so relevant treating services can share and access key clinical information. Given modern advances in technology, a shared electronic health record is a reasonable suggestion, with recent Australian efforts focused on developing a national ‘My Health Record’ system [34] and local examples including trials in remote Indigenous communities of the Ngaanyatjarra Lands in Western Australia [35] and metropolitan Brisbane [36]. However, complexity implementing electronic client data systems exists, such as technological issues and variation between services, managing the needs of multiple stakeholders, security concerns, high cost, varied computer literacy among staff and gaining clinician and client buy‐in to drive uptake [35, 36]. Overcoming these barriers is possible however, with carefully planned change management processes, adequate funding, strong leadership, effective governance and approaches designed to engage clients with complex needs [35, 36]. By clearly articulating to communities how the benefits outweighs risks to privacy may also enhance uptake [35]. Further, if health data related to Aboriginal people are being collected and shared, Indigenous Data Sovereignty must be considered to minimise risk of misuse and exploitation, and to ensure principles accessibility, ownership and Indigenous voice (among others) are upheld [37]. Taken together, these results indicate that the concept of developing a shared health record system to improve communication and referral between services has merit, however, it needs to be carefully planned, adequately resourced and designed with Aboriginal communities for a system to be ethical, feasible and useful.

Adoption of telehealth and videoconferencing was also identified as a useful way to increase engagement in rural areas. Although these approaches can overcome some barriers, such as distance, the disembodied nature of telehealth can make it harder to build rapport with clients and create enduring therapeutic relationships. A recent systematic review of reported outcomes of telehealth service among Aboriginal people found that telehealth enhanced clinical outcomes, facilitated greater access and that many Aboriginal people had positive telehealth experiences [38]. Further benefits arose from the opportunity to receive health services within a Community, rather than having to travel [38]. Although these approaches are exciting, ongoing disparities in access to stable and affordable internet connections can limit equitable access, particularly to those in rural areas [39]. These access barriers should also be considered, and face‐to‐face options should also be made available, where possible. Similarly to the notion of electronic health records, concerns about the privacy of telehealth are also documented [39], further demonstrating the necessity to develop both services in partnership with Communities to ensure they are culturally safe and functionality is clearly communicated.

### 4.4 Strengths and Limitations

This study only presented the views of ACCHOs and non‐ACCHO AOD or primary healthcare service providers who work with Aboriginal people in the NSW health services and did not explore those of clients or their support people, so further research that examines client perspectives is crucial to providing necessary insight into this subject. Due to resource limitations and our recruitment of services that we deemed suitable, we were unable to invite all relevant services across NSW to take part, potentially biasing recruitment. Although we had a small sample, meaning our findings are not representative of all rural NSW service providers, we included professionals from six services, ACDAN and we had extensive experience within our Aboriginal research lead who led analysis, providing the opportunity for complex ideas to develop.

## 5 Conclusion

There are opportunities to optimise AOD related services to Aboriginal people in rural NSW through expansion of programs that encourage standardised, culturally meaningful care that can be tailored to individual services, in both ACCHO and non‐Aboriginal services. ACCHOs and Aboriginal workers have key therapeutic skills that should be acknowledged and fostered through systemic changes that increase access to AOD training and better remuneration. Technological advances such as shared clinical records and telehealth may provide opportunities to improve service delivery; however, both need to be designed with Aboriginal communities and in a culturally safe way.

## Author Contributions

Raechel Wallace led data collection and analysis. Sara Farnbach conceptualised the study with Anthony Shakeshaft. Simon Clay and Sara Farnbach contributed to data analysis and manuscript development. All authors reviewed, commented on and approved the manuscript.

## Conflicts of Interest

The authors declare no conflicts of interest.

## Data Availability

The data that support the findings of this study are available on request from the corresponding author. The data are not publicly available due to privacy or ethical restrictions.
